# From Lake Victoria to the Tap: Antibiotic Resistance and Pathogenic Contamination of Kisumu City Water Supply and Wastewater Network

**DOI:** 10.1111/tmi.70105

**Published:** 2026-02-16

**Authors:** Oleg N. Reva, Anthony Sifuna, Francis Orata, Caroline Omolo, Jacob Stanley Iramiot, Mark C. Enright, Awelani Mutshembele, Jian Zhou, William A. Shivoga

**Affiliations:** ^1^ Department of Biochemistry, Genetics and Microbiology, Centre for Bioinformatics and Computational Biology University of Pretoria Pretoria South Africa; ^2^ Department of Medical Biochemistry Masinde Muliro University of Science and Technology Kakamega Kenya; ^3^ Department of Pure and Applied Chemistry Masinde Muliro University of Science and Technology Kakamega Kenya; ^4^ Water and Sanitation Company (KIWASCO) Kisumu Kenya; ^5^ Department of Microbiology and Immunology Busitema University, Faculty of Health Sciences, Mbale Campus Mbale Uganda; ^6^ Manchester Metropolitan University Manchester UK; ^7^ South African Medical Research Council‐Office of AIDS and TB (SAMRC‐OATB) Pretoria South Africa; ^8^ Department of Biological Sciences, Centre of Excellence for Water and Environment Resources Management Masinde Muliro University of Science and Technology Kakamega Kenya

**Keywords:** antibiotic resistance genes, metagenomics, pathogen surveillance, wastewater, water microbiome, waterborne diseases

## Abstract

Waterborne diseases and antimicrobial resistance (AMR) pose mounting public health threats across sub‐Saharan Africa, particularly in rapidly urbanising regions dependent on untreated or poorly treated surface waters. This study applied shotgun metagenomic sequencing to characterise microbial communities, virulence factors and antibiotic resistance genes (ARGs) in water samples collected from Lake Victoria, River Wigwa, Dunga Water Treatment Plant, Nyalenda Wastewater Stabilisation Ponds and the tap water outlet in post‐treatment supply pipe in Kisumu city (Kenya). Bacterial taxa dominated all metagenomes, with 121 classes represented. Cyanobacteria, particularly *Planktothrix*, were highly abundant in lake and tap water, whereas wastewater and river samples exhibited greater taxonomic diversity. Major human pathogens, including *Pseudomonas aeruginosa* , *Klebsiella pneumoniae* , *Escherichia coli* , *Acinetobacter baumannii* and *Bacillus cereus/anthracis*, were detected in nearly all samples, with unexpectedly high prevalence in tap water. Viral indicators of faecal contamination (adenoviruses, enteroviruses and torque teno viruses) corroborated widespread wastewater influence. Functional gene profiling revealed a rich resistome comprising aminoglycoside‐modifying enzymes, β‐lactamases, vancomycin‐resistance operons and disinfectant‐resistance determinants. The highest ARG and virulence gene frequencies occurred in tap and treatment‐plant water, suggesting that incomplete disinfection and biofilm persistence promote the proliferation and exchange of ARGs between environmental and pathogenic taxa. In contrast, Lake Victoria water exhibited lower ARG abundance, reflecting natural self‐purification processes. These findings underscore the inadequate water treatment and open wastewater systems create ecological ‘hotspots’ for ARG selection and horizontal gene transfer. Metagenomic surveillance integrated into One Health frameworks can enhance risk forecasting and guide interventions to mitigate AMR emergence and dissemination in freshwater systems serving over 35 million people across the Lake Victoria basin.

## 1 Introduction

Waterborne diseases remain a major public health challenge in sub‐Saharan Africa, where many communities rely on untreated surface water [1, 2]. In Kisumu City, on the northeastern shore of Lake Victoria, rapid urbanisation, poor sanitation, and frequent flooding cause recurrent microbial contamination. The lake is especially vulnerable due to ageing municipal infrastructure and dependence on untreated surface sources. Traditional culture‐based monitoring offers limited insight into microbial and resistance diversity, whereas metagenomic sequencing allows simultaneous detection of pathogens, virulence and resistance genes—an essential tool for integrated One Health risk assessment [3, 4, 5, 6, 7]. This study aimed to characterise the microbial and resistome landscapes of natural and industrial water reservoirs in Kisumu and nearby peri‐urban settlements to strengthen surveillance and risk assessment frameworks.

The Lake Victoria basin exemplifies these challenges [8]: despite improvements in sanitation and public health, untreated wastewater continues to pose serious risks [9]. In a globalised world, pathogen spread makes Africa's antimicrobial resistance (AMR) crisis a shared international responsibility. The region remains a hotspot for waterborne disease outbreaks [10] and multidrug resistance (MDR) evolution [11], as seen in the 2022–2023 cholera outbreak that began in Malawi and spread to South Africa [12, 13].

Studies from the Kakamega County General Teaching and Referral Hospital (KCGTRH) in western Kenya revealed high MDR rates among pathogenic isolates, including locally evolved and imported strains [14, 15, 16]. These involve both well‐known pathogens and emerging ‘X‐pathogens’ opportunistically acquiring multidrug resistance [17, 18].

Addressing AMR and waterborne infections requires a One Health approach recognising links between human, animal, and environmental systems [19]. In aquatic ecosystems such as Lake Victoria, wastewater discharge, livestock runoff and human water use promote ARG and mobile element exchange [20, 21]. Environmental reservoirs act as long‐term ARG sinks that can re‐enter clinical settings via water or food chains [22]. Hence, integrating environmental metagenomic surveillance into One Health frameworks is vital for early pathogen and resistance detection [23]. Global initiatives by WHO, FAO, and UNEP emphasise coordinated data sharing and joint risk assessment at the human–animal–environment interface [24]. A significant level of faecal contamination of water at the point of consumption in Kisumu revealed by isolation of thermotolerant coliforms, suggesting piped water continues to be exposed to water contamination, was reported previously [25].

Accordingly, this study conducted metagenomic analyses of water samples from natural reservoirs, water supply systems, and wastewater networks in Kisumu City (Kenya) to evaluate contamination by potentially pathogenic, antibiotic‐resistant, and virulent microorganisms, complementing prior work demonstrating the power of metagenomics in outbreak risk prediction [6, 7].

## 2 Methods

### 2.1 Sample Collect**i**on, Environmental DNA Extraction and Sequencing

Water samples were collected from the shores of Lake Victoria, the Wigwa River and municipal reservoirs in Kisumu City (Table 1; see the map of sampling points in Figure S1). The sampling points represent a water circulation system within the Kisumu municipality that includes natural water bodies (the lake and the river), water uptake, treatment and supply facilities, and a wastewater stabilisation pond.

**TABLE 1:** General characterisation of the samples.

Sample	Description	Abbreviated sample name	Total number of reads	Unidentified reads (%)
D001	Tap water collected from post‐treatment supply pipe at the treatment plant operated by the Kisumu Water and Wastewater Company	Tap water	16,583,636	50.5
D003	Surface water collected at Hippo Point recreational lakeside area near the city water intake facility on Lake Victoria	Lake Victoria	7,600,379	56.3
D004	Surface water collected at the mouth of the Wigwa River where it enters Lake Victoria	River Wigwa	5,076,223	53.2
D005	Wastewater sample collected from a stabilisation pond at the Kisumu Wastewater Recycling Centre	Wastewater	20,594,224	55.7
D006	Water collected from the sedimentation tank at the water treatment plant operated by the Kisumu Water and Sanitation Company	Treated water	16,842,931	48.2

All water samples for metagenomic analysis were collected on 23 October 2023 between 10:00 AM and 3:00 PM. Samples were taken at the end of the short autumn rain season which was modestly wet in October 2023 with 15.8 mm rainfall in a week before sampling. The temperature at the time of sampling was ~30°C that is within the expectation for the season.

All sampling activities were conducted with the knowledge and permission of the relevant local authorities responsible for water supply and sanitation infrastructure, including access to municipal treatment and distribution facilities. The study involved environmental water samples only and did not include human participants, personal data, or clinical specimens; therefore, formal human subject ethical approval was not required. Sampling and data generation were performed in accordance with institutional research guidelines, national regulations, and international best practices for environmental and public health research, ensuring responsible use of data for public interest and policy development.

Each 500 mL sample was taken in sterile HDPE Griffchem bottles (China), lowered ~30 cm below the surface in natural or industrial water bodies (lake, river, wastewater ponds, or treatment plant basins). Drinking water was collected from the post‐treatment supply pipe after a 2‐min flush.

Samples were filtered through sterile 0.45 μm CN Gridded membranes (Nantong FilterBio, China) using a Rocker 900 vacuum system (Rocker Scientific, Taiwan). Filters were cut into sterile 1.5 mL ZR BashingBead Lysis Tubes (Zymo Research, USA), vortexed for 30 min, and processed per the manufacturer's protocol. Prior to submission for sequencing, DNA quantity was measured using a Qubit 4 Fluorometer (Thermo Fisher Scientific, USA) to ensure a total input of ≥ 200 ng, and DNA integrity was assessed on 1% agarose gels stained with ethidium bromide and visualised using a UVP Benchtop UV transilluminator (UVP, USA). Metagenomic DNA library preparation, including library molarity normalisation, and sequencing were performed by Novogene (Beijing, China) on the NovaSeq PE150 platform.

### 2.2 Bioinformatic Analysis

Raw NovaSeq paired‐end reads were quality‐trimmed with Trimmomatic v0.36 [26] (Phred33) using parameters ILLUMINACLIP:TruSeq3‐PE.fa:20:30:10, LEADING:3, TRAILING:3, SLIDINGWINDOW:4:15, and MINLEN:50. Reads were scanned in 4‐base windows, trimmed when the mean quality dropped below 15, adapters removed, and reads < 50 bp discarded. Raw reads are deposited in the NCBI SRA BioProject PRJNA1345882.

Quality‐trimmed reads were taxonomically classified using Kaiju v1.6.2 [27] (NR + eukaryote database, greedy mode) with the program‐run options set by default, producing TSV summaries imported into MEGAN v6.25.10 [28] for visualisation. The results of taxonomic binning were organised into tables at different taxonomic ranks, summarising the numbers of reads assigned to the same genera, families, and higher‐level taxa. In these tables, read counts assigned to lower taxonomic levels were aggregated into higher‐level taxa, whereas reads assigned above the selected taxonomic level were ignored. Alpha‐diversity parameters were calculated from raw taxon counts, whereas for beta‐diversity comparisons between samples, taxon abundance counts were normalised using the centred log‐ratio (CLR) transformation with zero handling [29] implemented in the scikit‐bio v0.7.1.post1 Python module.

Alpha‐diversity parameters, Euclidean distance matrix and principal component analysis (PCA) were calculated using functions of the program PAST 4.02 [30].

Metagenomic assembly was performed with metaSPAdes v3.15.0 [31], retaining contigs ≥ 500 bp and ≥ 10× coverage. Gene prediction and annotation used Prokka v1.14 [32], generating GenBank‐compatible files.

ARG‐containing contigs (5 kbp fragments) were aligned against the NCBI nt database using BLASTn (megablast) with default settings. ARGs were identified with RGI v6.0.5 [33] against the Comprehensive Antibiotic Resistance Database (CARD), and virulence factors with Abricate v1.0.1 against the Virulence Factor Database (VFDB) (https://github.com/tseemann/abricate). Outputs were visualised using an in‐house Python3 (matplotlib v3.10.1) script, available with data at Zenodo (https://zenodo.org/records/17406069).

All analyses were performed on the Centre for Bioinformatics and Computational Biology (CBCB) high‐performance computing cluster, University of Pretoria, South Africa (http://wiki.bi.up.ac.za/).

## 3 Results

### 3.1 Microbial Community Distribution

**FIGURE 1:**
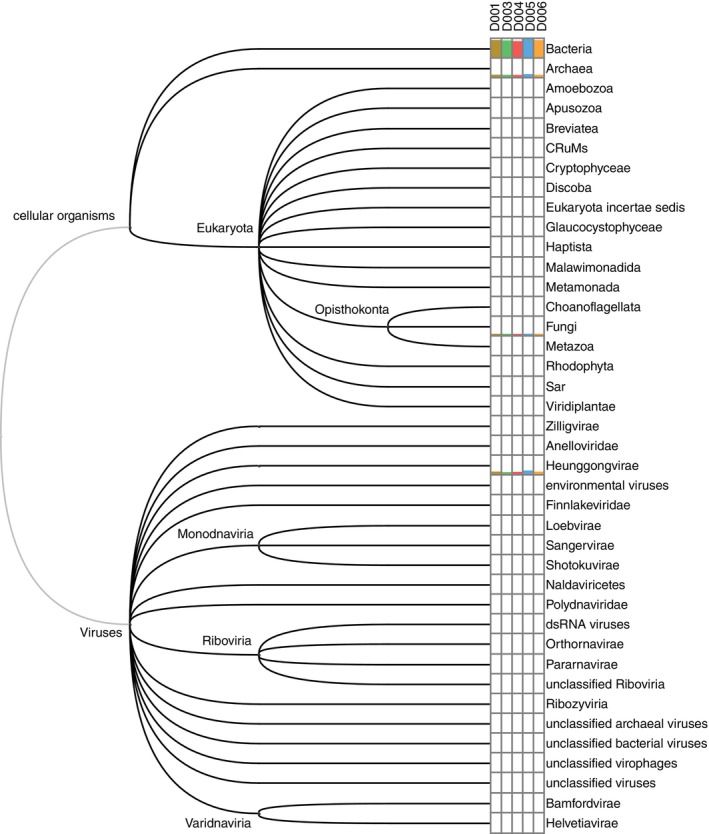
Taxonomic phyla of organisms identified in five water samples. Colour bars show abundance of DNA reads binned to the respective phyla. The tree was designed by Megan 6.25.10 based on phylogenetic relations predicted in the NCBI taxonomy database.

Across all sites, bacterial taxa dominated the metagenomic assemblages (Figure 1). This dominance can be attributed to the specificity of the DNA extraction kits, which are optimised for isolating DNA from bacterial cells. Nevertheless, a substantial proportion of the generated reads were assigned to archaea, green and red algae, protists, fungi, and viruses.

Alpha‐diversity metrics calculated for different samples based on taxonomic binning at the genus level are shown in Table 2. While the numbers of genera identified in the different samples are similar, the samples differ markedly in taxonomic dominance, which affects the diversity parameters. The two samples from the water supply facility, the tap water sample (D001) and the sedimentation tank sample (D006), were characterised by the highest dominance and the lowest diversity, whereas the wastewater stabilisation pond sample (D005) showed the lowest dominance and the highest taxonomic diversity.

**TABLE 2:** Lower‐upper 95%‐confidence values of alpha‐diversity parameters calculated for metagenomic samples.

Metric^a^	Samples				
	D001	D003	D004	D005	D006
Taxa_S	2828	2820	2810	2856	2831
Chao‐1	2833–2859	2825–2847	2819–2845	2859–2883	2836–2859
Fisher_alpha	279.9	317.2	329.2	288.4	278.4
Dominance_D	0.437–0.438	0.068	0.186–0.188	0.01	0.457–0.458
Simpson_1‐D	0.5618–0.5627	0.9318–0.9324	0.8123–0.8136	0.9899	0.5424–0.5433
Shannon_H	2.743–2.749	5.254–5.261	4.414–4.424	6.092–6.095	2.685–2.69
Evenness_e^H/S^	0.0055	0.0678–0.0683	0.0294–0.0297	0.1548–0.1553	0.0051–0.0052
Berger‐Parker	0.6606–0.6613	0.2537–0.2548	0.4303–0.4319	0.0661–0.0665	0.6754–0.6761

^a^ Berger‐Parker, dominance index defined as the relative abundance of the most abundant taxon; Chao‐1, estimated genus richness; Dominance_D, Simpson dominance index; Evenness_e^H/S^, Shannon‐based evenness of distribution of taxa; Fisher_alpha, parameter of Fisher's log‐series distribution reflecting richness independent of sample size; Shannon_H, Shannon diversity (entropy) index; Simpson_1‐D, Simpson diversity index; Taxa_S, observed taxon (genus) richness.

Principal component analysis (PCA) and the Euclidean distance matrix calculated after CLR normalisation of read counts assigned to different genera (Figure S2A,B) showed that all samples had distinguishable microbiota compositions, with sample D005 from the wastewater stabilisation pond serving as an outgroup.

The most abundant microorganisms were cyanobacteria of the genus *Planktothrix*. DNA reads assigned to this taxon constituted approximately 67% of all reads in samples D001 and D006 (tap water and treated water), 41% in the environmental sample from the River Wigwa (D004), and 25% in the Lake Victoria water sample (D003), but nearly disappeared in the wastewater stabilisation pond (D005–0.1%), probably due to oxygen depletion. The high abundance of this genus in the metagenomic samples explains the extreme dominance metric reported in Table 2, as well as the marked decrease in dominance observed in sample D005.

**FIGURE 2:**
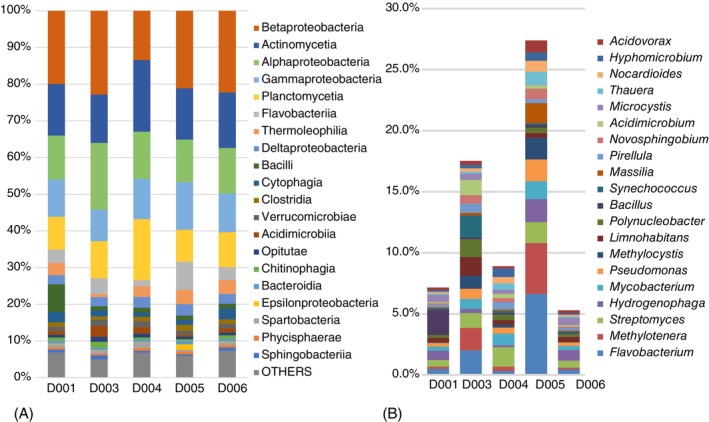
The result of binning the metagenomic reads to bacterial taxa. (A) Relative frequencies of the 20 most abundant bacterial classes. (B) Percentages of the 20 most abundant bacterial genera excluding the dominant *Planktothrix* spp.

The bacterial component of the microbiomes showed substantial diversity. The identified microorganisms belonged to 121 bacterial classes, 2880 genera (Table S1). The 20 most abundant classes (excluding Cyanobacteria) are shown in Figure 2A. The distribution of bacterial taxa was similar across all samples, including the tap water sample (D001), with Betaproteobacteria being the most abundant group, followed by Actinomycetia, Alphaproteobacteria, Gammaproteobacteria, Planctomycetia, and Flavobacteria. Sample D004 from the River Wigwa exhibited the highest diversity, with a marked decrease in Betaproteobacteria and increased representation of Actinomycetia and Planctomycetia. The tap water sample (D001) was enriched with spore‐forming Bacilli.

Water chlorination at the treatment point likely eliminates most unicellular eukaryotes, resulting in water enriched with bacterial species. The 20 most abundant bacterial genera contributed 27.4% of the total taxonomic diversity in the wastewater sample (D004), whereas in the other samples their contribution dropped to 5.3%–17.5% (Figure 2B). The most abundant bacterial genera were *Flavobacterium*, *Methylotenera*, *Bacillus*, *Hydrogenophaga* and *Methylocystis*. The striking abundance of methane‐ and hydrogen‐oxidising bacteria, which are typically associated with wetlands and lake sediments, even in the tap water, suggests substantial contamination with organic matter causing oxygen depletion.

### 3.2 Detection of Pathogens of Public Health Concern

A considerable level of contamination with household and agricultural wastewater was expected in this region. To evaluate the risks posed by such contamination, we screened microorganisms and viruses that serve as indicators of faecal pollution or are known to cause disease outbreaks. Table 3 presents the counts and relative abundances of potentially pathogenic bacterial species identified in the metagenomic samples. Of particular concern was the detection of various pathogens in the tap water sample, including *Pseudomonas aeruginosa* , *Klebsiella pneumoniae* , *Escherichia coli* (including *Shigella* spp.), the *Bacillus cereus/anthracis* group, *Acinetobacter baumannii* , *Salmonella enterica* , *Listeria monocytogenes* , *Legionella pneumophila* , *Vibrio cholerae* and *Yersinia pestis* , often in titers even higher than in other samples, including the wastewater treatment pond (Table 3). Tap water and water from the treatment facility also showed elevated levels of faecal indicators such as *Enterococcus faecalis* and *Enterococcus faecium* , reflecting possible contamination with wastewater.

**TABLE 3:** Frequencies of potentially pathogenic bacterial species identified by Kaiju taxonomic read binning.

Species	Absolute no. reads					Percentages from total no. reads				
	D001	D003	D004	D005	D006	D001	D003	D004	D005	D006
*Acinetobacter baumannii*	1130	383	272	1402	746	0.013779	0.011537	0.011445	0.015382	0.008558
*Bacillus anthracis*	1963	57	69	57	414	0.023937	0.001717	0.002903	0.000625	0.004750
*Bacillus cereus*	112,119	274	212	1132	373	1.367193	0.008254	0.00892	0.012419	0.000034
*Brucella suis*	3	3	3	2	3	0.000037	0.000090	0.000126	0.000022	0.000034
*Enterococcus faecalis*	1513	132	41	204	461	0.018450	0.003976	0.001725	0.002238	0.005289
*Enterococcus faecium*	8116	97	74	394	1612	0.098968	0.002922	0.003114	0.004323	0.018494
*Escherichia coli*	1448	702	489	3164	1255	0.017657	0.021146	0.020576	0.034713	0.014398
*Klebsiella pneumoniae*	2534	819	610	1548	2548	0.030900	0.024670	0.025667	0.016983	0.029232
*Legionella pneumophila*	238	229	147	934	252	0.002902	0.006898	0.006185	0.010247	0.002891
*Listeria innocua*	32	0	1	10	0	0.000390	0.0	0.000042	0.000110	0.0
*Listeria monocytogenes*	558	77	53	341	134	0.006804	0.002319	0.002230	0.003741	0.001537
*Neisseria meningitidis*	116	60	62	304	123	0.001415	0.001807	0.002609	0.003335	0.001411
*Pseudomonas aeruginosa*	1758	1081	782	4120	1906	0.021437	0.032562	0.032904	0.045201	0.021867
*Salmonella enterica*	20	0	0	1	10	0.000244	0.0	0.0	0.000011	0.000115
*Shigella flexneri*	15	3	4	26	12	0.000183	0.000090	0.000168	0.000285	0.000138
*Staphylococcus aureus*	4	0	1	1	0	0.000049	0.0	0.000042	0.000011	0.0
*Staphylococcus epidermidis*	134	7	9	37	18	0.001634	0.000211	0.000379	0.000406	0.000207
*Stenotrophomonas maltophilia*	988	847	519	2974	999	0.012048	0.025514	0.021838	0.032628	0.011461
*Streptococcus equi*	11	9	8	40	22	0.000134	0.000271	0.000337	0.000439	0.000252
*Streptococcus pneumoniae*	3576	106	84	408	127	0.043606	0.003193	0.003534	0.004476	0.001457
*Streptococcus suis*	461	106	105	299	530	0.005621	0.003193	0.004418	0.003280	0.006080
*Vibrio cholerae*	211	258	93	626	212	0.002573	0.007772	0.003913	0.006868	0.002432
*Yersinia pestis*	12	15	7	24	11	0.000146	0.000452	0.000295	0.000263	0.000126

While bacteriophages were prevalent in the water samples, many viruses associated with humans and animals were also detected (Table 4). Several of these viral taxa, such as adenoviruses, enteroviruses, torque teno viruses and astroviruses, have been reported as suitable markers of faecal contamination in water sources [34, 35]. The highest level of faecal contamination was observed in the wastewater stabilisation ponds, as expected. However, the tap water and water treatment facility samples were also significantly contaminated, showing higher viral loads than the environmental samples from the River Wigwa and Lake Victoria.

**TABLE 4:** Counts of reads binned to viral taxa associated with human diseases and serving as faecal contamination markers.

Viral taxa	D001	D003	D004	D005	D006	Indicators and disease causative agents
Adenoviruses	7	6	6	27	15	WHO‐recommended viral indicators of faecal pollution (Mastadenovirus, Atadenovirus, Siadenovirus, Aviadenovirus)
Enteroviruses	7	3	2	11	4	Faecal indicators (Picornaviridae, includes polioviruses, coxsackieviruses, echoviruses)
Torque teno viruses	5	3	1	6	6	Human faecal biomarker (Alpha‐, Beta‐ and Lambda‐torqueviruses, Iotatorqueviruses)
Astroviruses	0	1	1	3	1	Faecal indicators, gastroenteritis in children (Mamastrovirus)
Noroviruses	1	3	0	3	0	Leading cause of viral gastroenteritis (Caliciviridae)
Tulane viruses	29	3	10	37	12	A recommended surrogate for norovirus control (Caliciviridae)
Rotavirus	0	2	0	10	0	Major cause of infantile diarrhoea (Reoviridae)
Heunggongvirae	15	10	6	33	16	Causative agents of many infectious diseases

Another concern was the detection of noroviruses, Tulane viruses, and rotaviruses at high titers in all samples, including tap water, although to a lesser extent in the Lake Victoria sample. These viruses are leading causes of gastroenteritis, especially in children. Their presence also indicates substantial contamination of regional water resources with wastewater from households, clinics, and farms.

A significant proportion of metagenomic reads was assigned to Heunggongvirae—a realm of negative‐sense single‐stranded RNA (ssRNA) viruses. This group includes the causative agents of many severe diseases, such as influenza, Ebola, Lassa, measles, and rabies. These results, however, should be interpreted with caution, as RNA viruses cannot directly contribute to DNA reads generated from metagenomic samples. The detected viral sequences may originate from integrated cDNA fragments within host genomes or from stable viral cDNA derived from decayed host cells; however, false predictions arising from misalignment of short DNA fragments should not be excluded. Regardless of their source, the relative abundance of these reads was highest in the wastewater pond, tap water, and water treatment facility, and lowest in the Lake Victoria sample (Table 4).

### 3.3 Antimicrobial Resistance and Virulence Genes

Illumina DNA reads generated from the different samples were assembled into contigs using the SPAdes assembler [31]. Contigs shorter than 500 bp and those with coverage lower than 10× were excluded from further analysis. Protein‐coding genes within the contigs were predicted using Prokka. Statistical parameters of the assembled contigs are presented in Table 5.

**TABLE 5:** Statistical parameters of the assembled contigs.

Sample	No. contigs	Total length	Percentage of assembled reads (%)	Maximal contig lengths (bp)	Median contig length (bp)	L50	No. predicted protein coding genes
D001	6585	21,043,981	34.85	695,354	1221	416	20,240
D003	26,370	34,718,541	25.21	59,410	860	5332	37,504
D004	4120	13,049,257	60.42	49,072	1521	523	11,522
D005	31,827	69,372,325	46.76	172,172	1218	4749	75,800
D006	5156	12,388,658	74.63	93,579	1173	643	10,997

**FIGURE 3:**
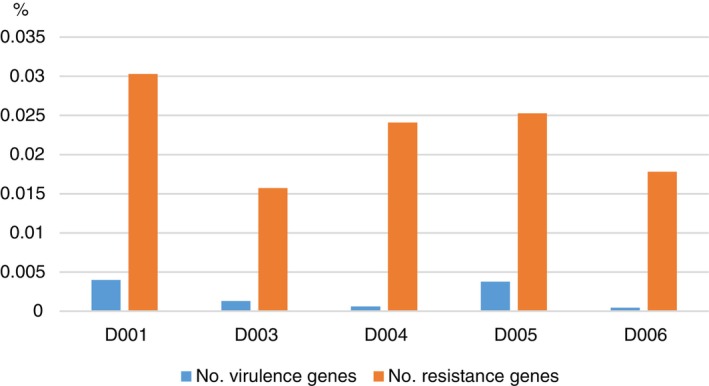
Percentages of genes associated with virulence and antibiotic resistance in different samples in relation to the total numbers of predicted protein‐coding genes.

ARGs were identified searching through the CARD database using the program RGI. VFs were detected searching through the VFDB database using the program Abricate. The relative frequencies of virulence and antibiotic resistance genes in the different samples are shown in Figure 3. Remarkably, the highest frequencies of both gene categories were observed in the tap water, followed by the wastewater stabilisation ponds and the river water.

**FIGURE 4:**
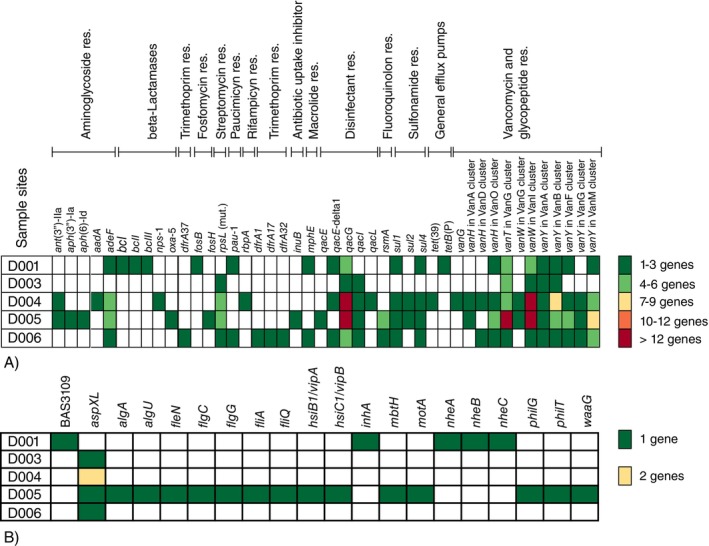
Distribution of (A) antibiotic resistance genes (ARGs) grouped by affected antibiotics; and (B) virulence associated genes in contigs assembled from metagenomic samples. Cell colour depicts number of genes found in the sample as explained in the figure legend.

A wide range of ARGs and VFs related to adhesion, toxin production, and iron acquisition was detected (Figure 4A,B, respectively). Aminoglycoside‐modifying enzymes, β‐lactamases, vancomycin‐resistance genes, and disinfectant‐resistance determinants, including *tet*‐family efflux pumps, were the most abundant categories across the samples. The resistome profiles revealed substantial ARGs diversity, reflecting varying environmental and anthropogenic inputs within the region.

Genes encoding enterotoxins (*nheABC*), the immune inhibitor A metalloprotease (*inhA*), and antibiotic resistance determinants, including vancomycin‐resistance genes (*vanH*, *vanT*, *vanW* and *vanY*), β‐lactamases (*bcI*, *bcII* and *bcIII*), and several other resistance genes of different categories, were detected in the tap water sample. These genes were co‐located on a few contigs originating from the *Bacillus cereus/anthracis* lineage. Another contig of the same origin carried BAS3109 anthrolysin, a marker specific to *B. anthracis* [36]. The abundance of virulence and antibiotic resistance factors derived from the *B. cereus/anthracis* lineage correlates with the increased frequency of reads associated with the genus *Bacillus* in this sample (Figure 2B). Macrolide and disinfectant resistance genes of the *mphE* and *qac* families found in the same sample have originated from genomes and virulence plasmids of enterobacteria (*Escherichia*, *Proteus* and *Salmonella*). The paucimicin‐resistance gene *pau*‐1 came from an unclassified betaproteobacterium, whereas the source of the contig carrying the *adeF* gene could not be identified.

Metagenomic samples from the river, wastewater stabilisation pond, and the treatment plant reservoir revealed an exceptional diversity of antibiotic resistance genes originating from waterborne pathogens (*Escherichia*, *Klebsiella*, *Salmonella*, *Proteus*, *Legionella*), clinical pathogens (*Staphylococcus aureus, Streptococcus*, and *Mycobacterium tuberculosis* ), opportunistic pathogens ( *Pseudomonas aeruginosa* , *Burkholderia*, *Xanthomonas, Stenotrophomonas, Acinetobacter, Ricketsiella*) and environmental as well as conditionally pathogenic bacteria (*Acidovorax, Actinomadura, Actinoplanes, Actinomycetes, Aeromicrobium, Agrobacterium, Aquihabitans, Aquipseudomonas, Blastochloris, Bosea, Cloacibacter, Conexibacter, Deinococcus, Dermatobacter, Diaphorobacter, Dickeya, Flavobacterium, Hydrogenophaga, Jannaschia, Kitasatospora, Luteolibacter, Marichromatium, Megasphera, Methylobacterium, Methylococcus, Microbacterium, Microcella, Mycolicibacterium, Myxococcus, Phenylobacterium, Pimelobacter, Rhodobacter, Roseateles, Sediminicoccus, Solirubrobacter, Solwaraspora, Tautonia, Thauera, Thiomonas, Variovorax*), cyanobacterium *Planktothrix* and archaeum *Halobacter*. In contrast to the tap water sample, *Bacillus* does not contribute to the resistome pools of other samples.

Multiple VFs detected in the wastewater sample (Figure 4B) were predominantly derived from *Pseudomonas aeruginosa* , except for *Brucella*'s *aspXL* acyl carrier protein, which plays a role in lipopolysaccharide (LPS) modification. Contigs originating from *Brucella* genomes carrying this gene were found in all samples except the tap water.

Lake Victoria water sample was characterised by the smallest number of ARGs and VFs, indicating a significant self‐purification potential of this ecological system. Antibiotic resistance genes were associated with the cyanobacterium *Planktothrix*, the predatory bacterium *Corallococcus*, *Xanthomonas* and the actinobacteria *Ilumatobacter* and *Aquihabitans*. All of these taxa are naturally characterised by the presence of multiple intrinsic antibiotic resistance genes.

## 4 Discussion

Our findings show that the water intake system, tap water and wastewater stabilisation ponds in Kisumu City near Lake Victoria contain diverse pathogens and ARGs, with distribution networks serving as hotspots for opportunistic pathogens and MDR emergence. This poses a serious public health risk given limited local treatment capacity [8, 9]. The co‐occurrence of VFs and ARGs within several contigs suggests the rise of highly pathogenic, treatment‐resistant strains. These results highlight the need for metagenomic‐based water monitoring, improved municipal infrastructure, and biofilm control, while emphasising Lake Victoria's dual role as a pathogen reservoir and dissemination route, reinforcing the need for coordinated regional surveillance.

The abundance of pathogens in both natural and industrial water reservoirs appear to result from a high level of faecal contamination, supported by the frequent detection of DNA fragments from *Escherichia coli* and viral faecal indicators [34, 35]: adenoviruses, enteroviruses and torque teno viruses, in all samples (Tables 2 and 3). Lake Victoria, however, demonstrates a considerable capacity for self‐purification: the frequencies of ARGs and VFs were markedly reduced compared with other samples, including the tap water sample. This apparent decline in pathogen load, reflecting natural self‐purification processes in open aquatic environments, may be attributed to the activity of numerous protists identified in the metagenomic profile (Figure 1), which contribute to microbial grazing and pathogen removal, as well as to additional mechanisms including dilution and hydrodynamic dispersion, sedimentation and adsorption of microorganisms onto particles, solar UV irradiation‐induced inactivation, predation by bacteriophages and predator bacteria (e.g., myxobacteria such as *Corallococcus* spp., which were abundant in the Lake Victoria sample), and competitive exclusion within native microbial communities. Together, these physical, biological and ecological processes reduce the persistence and infectivity of enteric pathogens in surface waters [37, 38]. However, natural environmental self‐purification processes cannot fully counteract intensive faecal contamination, as indicated by the presence of *E. coli* and other intestinal bacteria in lake water.

Nevertheless, although the concentrations of waterborne pathogens and other disease agents decrease within the lake, they remain sufficiently abundant to pose continued public‐health risks that are confirmed by many other studies [39, 40, 41, 42, 43]. A likely source of environmental and pathogenic bacteria in the water‐supply system is the persistent colonisation of pipes and reservoirs by biofilm‐forming microbial consortia [44]. Cracks and clogs in the ageing municipal water‐supply infrastructure create ideal conditions for biofilm formation, while suboptimal disinfectant concentrations within the system select for AMR.

The bacterial taxa contributing most strongly to the resistome pools across the sampled environments are summarised in Table 6.

**TABLE 6:** Summary of 10‐top bacterial species, which appeared the most frequently in MegaBLAST outputs among hits against ARG containing contigs in different samples.

Sample	Taxon	Number of times found
D001	*Bacillus thuringiensis*	201
	*Bacillus cereus*	183
	*Bacillus anthracis*	84
	*Pseudomonas aeruginosa*	82
	*Klebsiella pneumoniae*	62
	*Bacillus mycoides*	53
	*Bacillus wiedmannii*	33
	*Bacillus paranthracis*	31
	*Escherichia coli*	31
	*Bacillus tropicus*	28
D003	*Planktothrix agardhii*	25
	*Castellaniella ginsengisoli*	17
	*Nocardia seriolae*	11
	*Paracoccus yeei*	11
	*Rhodococcus ruber*	10
	*Pseudomonas citronellolis*	9
	*Brucella anthropi*	9
	*Cupriavidus gilardii*	7
	*Brucella intermedia*	7
	*Nocardia cyriacigeorgica*	6
D004	*Bradyrhizobium diazoefficiens*	214
	*Bordetella parapertussis*	81
	*Pasteurella multocida*	76
	*Mycobacterium intracellulare*	75
	*Klebsiella pneumoniae*	66
	*Ralstonia pseudosolanacearum*	61
	*Pseudomonas aeruginosa*	46
	*Stenotrophomonas maltophilia*	44
	*Burkholderia cenocepacia*	35
	*Acinetobacter baumannii*	29
D005	*Pseudomonas putida*	331
	MAG: uncultured *Solirubrobacterales bacterium*	153
	*Pseudomonas fulva*	145
	*Escherichia coli*	115
	*Klebsiella pneumoniae*	106

	*Pseudomonas asiatica*	89
	*Pseudomonas aeruginosa*	70
	*Pasteurella multocida*	68
	*Stenotrophomonas maltophilia*	63
*Enterococcus faecium*	43
D006	*Bordetella parapertussis*	83
	*Proteus mirabilis*	69
	*Pasteurella multocida*	65
	*Escherichia coli*	64
	*Coxiella burnetii*	41
	*Pseudomonas aeruginosa*	35
	MAG: uncultured *Solirubrobacterales bacterium*	34
	*Klebsiella pneumoniae*	34
	*Mycobacterium intracellulare*	33
	*Planktothrix agardhii*	25

Lake Victoria is the main water source for Kisumu City, with intake at Dunga near the Dunga Water Treatment Plant. Water undergoes filtration, coagulation, pH adjustment, and chlorination before distribution. The tap water sample was collected post‐treatment. Despite these processes, inadequate treatment and long‐term system contamination promote resistant and pathogenic bacteria. This was evident in our study, where ARG frequency increased in the treatment plant tank within hours after collection from Lake Victoria (compare samples D006 and D003, Figure 3). While the overall frequency of pathogens in the water slightly decreased after treatment, except for *Enterococcus faecalis* , *E. faecium* and *Bacillus anthracis* , which increased (Table 3), the relative abundance of ARGs rose markedly (Figure 4A). The main sources of ARGs in this sample were pathogens such as *Bordetella parapertussis* , *Proteus mirabilis* , *Pasteurella multocida* , *Escherichia coli* , *Coxiella burnetii* and *Pseudomonas aeruginosa* . *Mycobacterium intracellulare* indicates biofilm persistence within the water distribution system. The resistome pool became significantly enriched in water from the treatment plant (Figure 4A), indicating an enteric‐environmental gene exchange zones.

In contrast to the water sample collected at the treatment plant, where potentially pathogenic bacteria were the major ARG contributors, the major sources of ARGs in Lake Victoria water were the environmental taxa *Planktothrix agardhii* and *Castellaniella ginsengisoli* . The co‐occurrence of actinobacteria (*Nocardia*, *Rhodococcus*) and α−/β‐proteobacteria (*Paracoccus*, *Castellaniella*, *Cupriavidus*) in the Lake Victoria sample is typical of biofilm‐rich and nutrient‐variable aquatic environments, whereas the presence of *Brucella* spp. and *Cupriavidus gilardii* indicates possible zoonotic or anthropogenic input (e.g., livestock runoff).

Water from the Dunga treatment plant is pumped into a tower for household distribution. The tap sample, taken from the outlet pipe, reflects a microbial community adapted to the applied disinfectant levels that permit bacterial growth and biofilm formation. The tower environment favours the proliferation of the *Bacillus cereus* /*anthracis* group, likely originating from the herd of hippos living near the Dunga intake at Hippo Point. Detection of *Pseudomonas aeruginosa* and *Klebsiella pneumoniae* indicates wastewater influence, while *Escherichia coli* signals faecal contamination.

The ecosystem of the Wigwa River is influenced by runoff from agricultural fields, as indicated by the dominance of *Bradyrhizobium diazoefficiens*, a common inhabitant of root‐associated environments. However, the co‐detection of human and veterinary pathogens (*Bordetella*, *Pasteurella*, *Klebsiella*, and *Acinetobacter*) suggests mixed agricultural and anthropogenic contamination coming from the city's drainage network. This environment exhibits complex resistome exchange between soil and clinical bacterial species (Figure 4A).

The dominance of multiple *Pseudomonas* species (both environmental and clinical) characterises the wastewater stabilisation pond as a biofilm‐prone, oxygenated aquatic system with substantial organic pollution. *P. putida* and *P. fulva* are common bioremediators but also carry multidrug efflux systems, whereas *P. aeruginosa* contributes clinically relevant ARGs. The presence of *Enterococcus faecium* and *Klebsiella pneumoniae* reflects human or animal faecal contamination. The proximity of the wastewater stabilisation pond to the lake shore suggests that contamination of Lake Victoria cannot be excluded, particularly during flooding events or via aquatic birds such as ducks and marabou storks attracted to this dumping site.

Natural water reservoirs, together with the Kisumu city water supply and recycling systems, form an interconnected ecological network influenced by high human population density, poor sanitation, livestock and crop farming, and the region's rich fauna and flora. Inadequate water treatment, combined with the open design of wastewater stabilisation ponds, creates an optimal environment for the evolution of antibiotic‐resistant pathogens through ARG selection and horizontal gene transfer between pathogens and environmental microorganisms [45]. Although these evolutionary processes underlying AMR development and their associated public health hazards are well recognised [46], many aspects of the emergence and persistence of AMR pathogens in natural environments remain poorly understood.

Our study supports earlier reports that pathogenic and environmental species' contributions to a habitat's resistome do not necessarily correlate with their abundance [7, 47]. A correlation was observed only in the tap water sample, where the high abundance of *Bacillus cereus* likely explains its elevated ARG contribution. In other samples, taxa abundance and resistome contribution were uncoupled. For instance, in river water, *Bradyrhizobium* spp. represented just 0.0024% of the microbiome yet dominated the resistome, unlike in Lake Victoria or wastewater samples, where they constituted 0.0032% of the taxonomic diversity, but their resistome impact was lower. Likewise, although *Planktothrix* cyanobacteria dominated most samples, they were ARG‐enriched only in Lake Victoria surface water, while low‐ARG strains prevailed elsewhere.

While the presence of enterobacteria indicating faecal contamination of the environment was consistently detected in all samples and corroborated by the abundance of indicator viruses (Table 4), the abundance of ARGs in the genomes of these pathogens varied significantly among samples (Table 6), showing no correlation with the abundance of these pathogens in the microbiomes (Table 3). This incongruence between species abundance and their contribution to the resistome can be explained, to some extent, by differences in the concentrations of various antimicrobials and other stressors in the environment [48], by complex symbiotic and antagonistic interactions among community members involved in biofilm creation [49], and by the presence of habitat‐specific mobilomes (e.g., conjugative plasmids, mobile genetic cassettes, and lysogenic phages), which are more likely to transfer ARGs between certain bacterial species than others [14, 50, 51]. All these factors are of great practical importance for epidemiological forecasting and for assessing the risks of antibiotic resistance development and the emergence of new disease outbreaks caused by multidrug‐resistant ‘superbugs’ [52, 53, 54].

## 5 Conclusions

This metagenomic study shows that water sources in the Lake Victoria basin near Kisumu City act as potential routes for spreading pathogenic and antibiotic‐resistant bacteria. Reliable pathogen and ARG surveillance is urgently needed in rapidly urbanising sub‐Saharan regions with dense populations and inadequate sanitation, where conventional monitoring underestimates microbial risks. The detection of clinically relevant pathogens and high‐risk antibiotic resistance genes in treated and tap water identifies critical weaknesses in the municipal water treatment and distribution system. These findings provide actionable evidence for engagement with water and sanitation authorities, supporting improved disinfection, biofilm control, and integration of metagenomic surveillance into routine monitoring. The results also inform community risk communication and support incorporation of environmental metagenomics into national One Health strategies for early warning and control of enteric pathogens and antimicrobial resistance.

By integrating taxonomic, resistome, and virulome analyses, the study offers a comprehensive view of water‐associated health hazards while revealing limitations of classical metagenomic surveillance for water safety. These arise from seasonal and anthropogenic factors: rainfall, flooding, faecal and industrial contamination, and complex microbe‐host interactions. Addressing them requires an integrated One Health approach linking environmental and clinical monitoring.

This work provides a baseline snapshot of microbiomes and resistomes in Kisumu's water systems. Future planned work will expand it into time‐series analyses to track environmental and human influences on pathogen and ARG distribution in the Lake Victoria basin, a freshwater source for 30–35 million people.

## Funding

This work was funded by the UK Global Challenges Research Fund Networking Grant GCRFNGR8\1143 and the UK National Institute for Health and Care Research grant East Africa AMR STOP Project (NIHR163838).

## Disclosure

The authors have nothing to report.

## Conflicts of Interest

The authors declare no conflicts of interest.

## Supporting information


**Figure S1:** Geographical locations of sampling points: 1—tap water (sample D002); 3—Lake Victoria (sample D003); 4—river Wigwa (sample D004); 5—stabilisation pond (sample D005); 6—sedimentation tank at the water treatment plant (sample D006). The map was exported from the OpenStreetMap server (https://www.openstreetmap.org/copyright?utm_source=chatgpt.com) operating under the Open Data Commons Open Database Licence (ODbL). Photos used in this figure are from O. Reva's private collection.

**Figure S2:** (A) Principal Component Analysis (PCA) plot of sampled microbiomes: D001—tap water; D003—Lake Victoria; D004—river Wigwa; D005—stabilisation pond; D006—sedimentation tank at the water treatment plant. (B) Euclidian distances between sampled microbiomes. Both PCA plot and distance matrix were calculated based on taxonomic profiles of bacterial genera using the program Past 4.02. DNA read counts associated with different genera were normalised prior to analysis using the centred log‐ratio (CLR) transformation.

**Table S1:** Bacterial genera identified in metagenomic water samples.

## Data Availability

The data that support the findings of this study are openly available in NCBI at http://www.ncbi.nlm.nih.gov/bioproject/1345882, reference number PRJNA1345882.
