# Supplementary figures and images for: From Lake Victoria to the Tap: Antibiotic Resistance and Pathogenic Contamination of Kisumu City Water Supply and Wastewater Network
Source: Trop Med Int Health. 2026 Feb 16;31(4):547–59. doi: 10.1111/tmi.70105 (PMC13050616; doi:10.1111/tmi.70105)

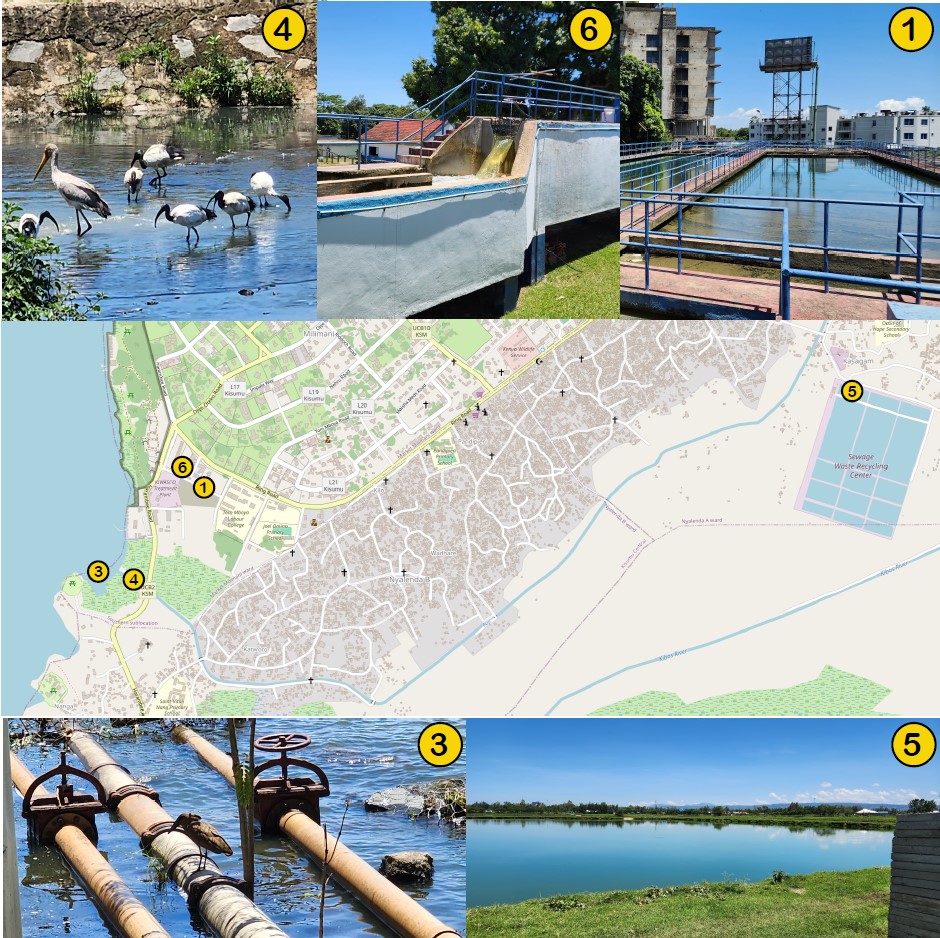

Supplement: Supplementary file 1 — Figure S1: Geographical locations of sampling points: 1—tap water (sample D002); 3—Lake Victoria (sample D003); 4—river Wigwa (sample D004); 5—stabilisation pond (sample D005); 6—sedimentation tank at the water treatment plant (sample D006). The map was exported from the OpenStreetMap server (https://www.openstreetmap.org/copyright?utm_source=chatgpt.com) operating under the Open Data Commons Open Database Licence (ODbL). Photos used in this figure are from O. Reva's private collection. [file TMI-31-547-s001.jpg]

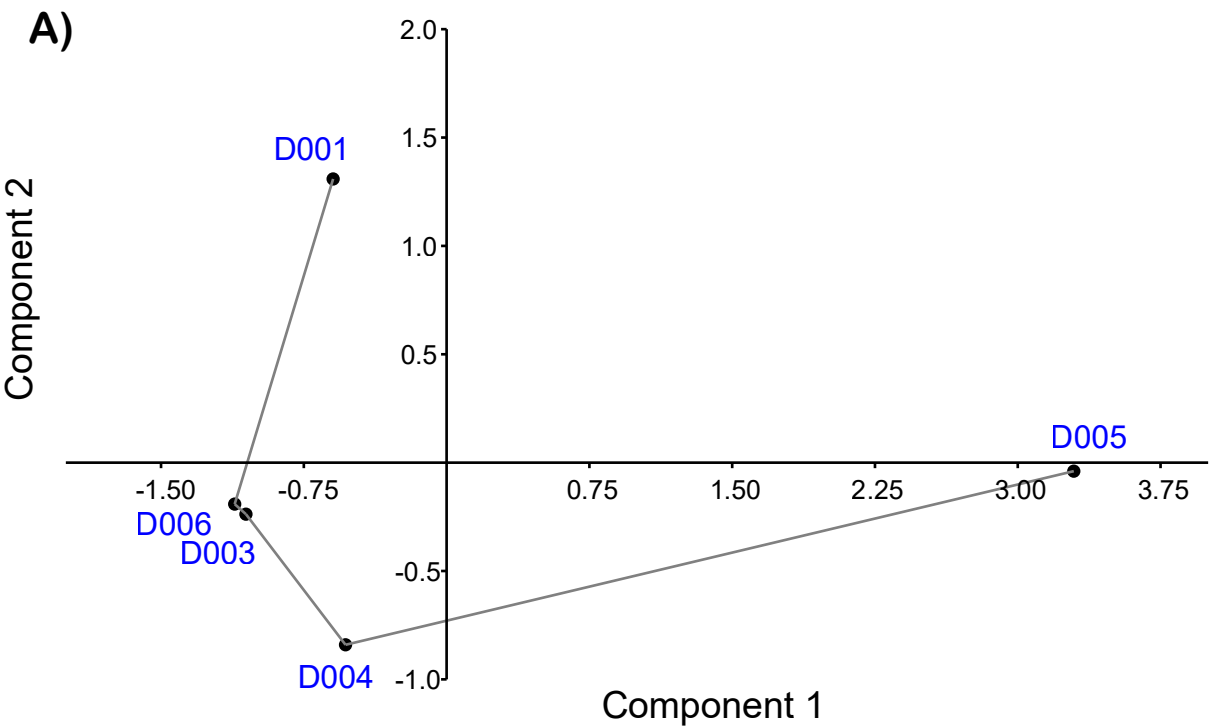

| <b>B)</b>   | <b>D001</b> | <b>D003</b> | <b>D004</b> | <b>D005</b> | <b>D006</b> |
|-------------|-------------|-------------|-------------|-------------|-------------|
| <b>D001</b> | 0.00        | 2.14        | 2.21        | 4.15        | 2.15        |
| <b>D003</b> | 2.14        | 0.00        | 1.98        | 4.51        | 2.16        |
| <b>D004</b> | 2.21        | 1.98        | 0.00        | 4.04        | 1.99        |
| <b>D005</b> | 4.15        | 4.51        | 4.04        | 0.00        | 4.55        |
| <b>D006</b> | 2.15        | 2.16        | 1.99        | 4.55        | 0.00        |

Supplement: Supplementary file 2 — Figure S2: (A) Principal Component Analysis (PCA) plot of sampled microbiomes: D001—tap water; D003—Lake Victoria; D004—river Wigwa; D005—stabilisation pond; D006—sedimentation tank at the water treatment plant. (B) Euclidian distances between sampled microbiomes. Both PCA plot and distance matrix were calculated based on taxonomic profiles of bacterial genera using the program Past 4.02. DNA read counts associated with different genera were normalised prior to analysis using the centred log‐ratio (CLR) transformation. [file TMI-31-547-s003.pdf]
